# Perceptions of healthcare workers on linkage between depression and hypertension in northern Ghana: a qualitative study

**DOI:** 10.1017/gmh.2024.86

**Published:** 2024-10-10

**Authors:** Dorothy Adu-Amankwah, Masih A. Babagoli, Raymond A. Aborigo, Allison P. Squires, Engelbert Nonterah, Khadija R. Jones, Evan Alvarez, Maria Anyorikeya, Carol R Horowitz, Benedict Weobong, David J. Heller

**Affiliations:** 1 Icahn School of Medicine at Mount Sinai, New York, NY, USA; 2 Navrongo Health Research Centre, Navrongo, Ghana; 3 Rory Myers School of Nursing at New York University, New York, NY, USA; 4 Arnhold Institute for Global Health, Icahn School of Medicine at Mount Sinai, New York, NY, USA; 5 Department of Population Health Science and Policy, Icahn School of Medicine at Mount Sinai, New York, NY, USA; 6 School of Global Health, York University, Toronto, ON, Canada

**Keywords:** depression, hypertension, healthcare workers, developing countries, community-based initiatives

## Abstract

Hypertension and depression are increasingly common noncommunicable diseases in Ghana and worldwide, yet both are poorly controlled. We sought to understand how healthcare workers in rural Ghana conceptualize the interaction between hypertension and depression, and how care for these two conditions might best be integrated. We conducted a qualitative descriptive study involving in-depth interviews with 34 healthcare workers in the Kassena-Nankana districts of the Upper East Region of Ghana. We used conventional content analysis to systematically review interview transcripts, code the data content and analyze codes for salient themes. Respondents detailed three discrete conceptual models. Most emphasized depression as causing hypertension: through both emotional distress and unhealthy behavior. Others posited a bidirectional relationship, where cardiovascular morbidity worsened mood, or described a single set of underlying causes for both conditions. Nearly all proposed health interventions targeted their favored root cause of these disorders. In this representative rural Ghanaian community, healthcare workers widely agreed that cardiovascular disease and mental illness are physiologically linked and warrant an integrated care response, but held diverse views regarding precisely how and why. There was widespread support for a single primary care intervention to treat both conditions through counseling and medication.

## Graphical abstract

**Figure:**
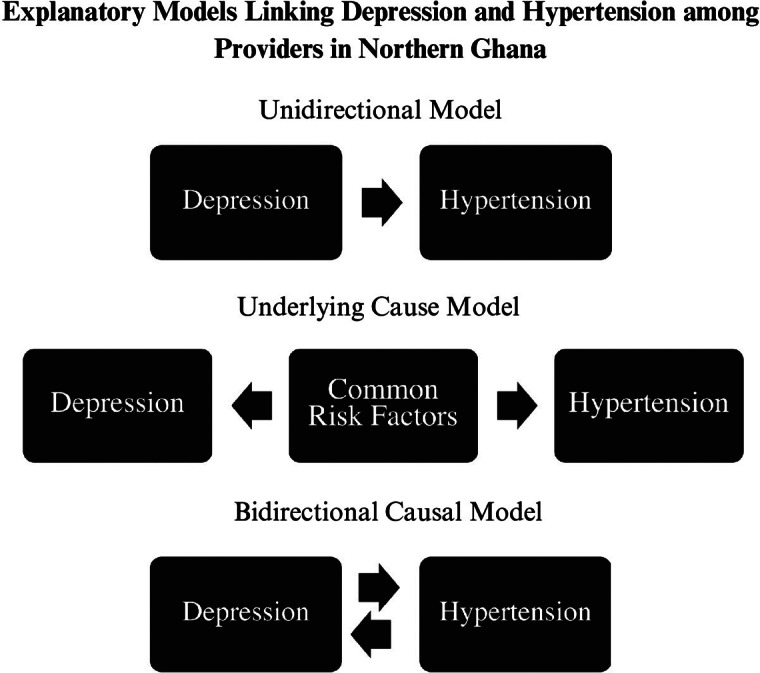

## Impact statement

As the global burden of disease rapidly shifts from acute and infectious diseases to chronic, noncommunicable conditions, health systems must adapt in developing the best primary care programs to manage these diverse diseases efficiently. Depression is now the world’s leading cause of disability, and high blood pressure and other heart diseases are leading causes of death. Yet even though these two diseases are common and severe, and often co-appear in the same patient, concurrent treatment for these two conditions is rare in low- and middle-income countries. Further, little is known about how frontline healthcare workers understand how these diseases interact. We conducted in-depth interviews with frontline nurse providers in Ghana’s national rural primary care program to understand how they feel these diseases interact causally and behaviorally – and whether they could be co-managed in their practice. We found broad consensus that depression and hypertension interact causally, and support for a single primary care model to treat both diseases at once, with differing views on its precise structure. Our results therefore not only support and inform the design and delivery of a program for integrated chronic disease care in rural Ghana, but may also inform and impact the adaptation and replication of such care models in other low-resource settings. As global demand rises for primary care programs that integrate mental health with other chronic disease control, these interventions must understand and respond to local needs and beliefs to succeed. Our findings offer a model for how to design these programs through community-level qualitative research. They suggest that the joint delivery of basic mental and cardiovascular primary health care may be both feasible and supported by frontline nurse providers where there is no doctor.

## Introduction

Chronic noncommunicable diseases (NCDs) cause 71% of deaths worldwide, and are the leading cause of global disability (Bigna and Noubiap, 2019). The incidence and prevalence of NCDs in low- and middle-income countries (LMICs) is rising as these nations’ disease profiles transition away from infectious conditions. Across sub-Saharan Africa (SSA) for example, rates of hypertension are climbing due to longer life expectancy and lifestyle changes (Boateng et al., 2015; Ademola et al., 2019; Haykin et al., 2020; Oduro et al., 2021). Mental illness, too, is increasingly common yet largely undiagnosed in SSA (Gbadamosi et al., 2022) and across LMICs. However this rising burden is often poorly captured due to social and cultural stigma (Mascayano et al., 2015; Tawiah et al., 2015). Globally, depressive disorder is the most common mental health disorder and the leading cause of disability (Friedrich, 2017; Endomba et al., 2020), while hypertension is the leading cause of premature death (Ngo et al., 2013; Boateng et al., 2015).

Ghana exemplifies this global trend. A 2021 study estimated hypertension prevalence in Ghana at 30.1% among females and 34.0% among males (Atibila et al., 2021). Rural areas, such as Ghana’s Upper East region, are not exempted, despite less exposure to Western dietary patterns and sedentary lifestyles. In 2017, about 25% of older adults in Ghana’s Upper East lived with hypertension, an increase of 5% from 2008 (Gómez-Olivé et al., 2017). Meanwhile, some 7% of adults 50 and older in Ghana live with depression, but rates as high as 39%–42% have been measured in select populations (Bahadur Thapa, 2014; Ademola et al., 2019).

Furthermore, multimorbidity of mental illness and other chronic conditions is rising in Ghana. Depression is the most common mental illness among individuals with NCDs, with global prevalence of 22%–23% (Boima et al., 2020). Depression and hypertension are especially comorbid, especially among vulnerable populations (Kretchy et al., 2014; Arokiasamy et al., 2015; Ademola et al., 2019; Geldsetzer et al., 2019; Boima et al., 2020; Endomba et al., 2020). A nationwide study in Ghana demonstrated that respondents reporting depression or unhappiness were more likely to have hypertension (Boateng et al., 2015). A study of depression among individuals with hypertension in Ghana reported an overall prevalence of 6.3%, including 8.4% of older adults (50+ years) (Boima et al., 2020), and a hospital-based study reported prevalence of depression as high as 42% among adults on antihypertensive medications (Ademola et al., 2019).

Evidence suggests a complex, bidirectional causal relationship between hypertension and depression (Boateng et al., 2015; Awuah et al., 2019; Endomba et al., 2020). In addition to physiological mechanisms, depressive symptoms may also lead to health behaviors and lifestyles that cause and exacerbate risk factors for hypertension (Stapelberg et al., 2011; Awuah et al., 2019). Conversely, risk factors for hypertension and cardiovascular disease such as sedentary lifestyle, alcohol and substance misuse may worsen depression (Bahadur Thapa, 2014; Adjaye-Gbewonyo et al., 2019; Awuah et al., 2019; Amu et al., 2021). Rates of disease control for both NCDs are poor in low-resource settings, due in part to inadequate physicians, including psychiatrists. Non-physician health workers, including nurses, pharmacists and community health workers (CHWs), can provide effective NCD care through behavior counseling and medication when there are physician shortages (Joshi et al., 2014; Heller et al., 2019; Patil et al., 2023).

In Ghana, which has fewer than one physician per 10,000 people, the Community-Based Health Planning and Services (CHPS) program provides high-quality primary healthcare services in rural areas such as the Upper East, through nurses and community health volunteers (Phillips, 2006; Haykin et al., 2020; Elsey et al., 2023). Nurses live and work in remote health outposts called CHPS compounds, operating as autonomous primary care providers, but also provide door-to-door care at patients’ homes. Volunteers offer health counseling at homes and compounds. CHPS treats many primary health conditions but provides no NCD care at compounds or homes, despite evidence that non-physicians can screen and treat hypertension and depression with similar efficacy to doctors (Abegunde et al., 2007; Patel et al., 2017; Heller et al., 2019; Laar et al., 2019). An effective CHPS intervention to treat NCDs must emphasize the local behavioral mechanisms by which these two diseases exacerbate each other. (Heller et al., 2019; Haykin et al., 2020; Wood et al., 2021; Patil et al., 2023). This study therefore used qualitative interviews to understand the perceptions of CHPS nurses and CHWs on how hypertension and depression interact causally and behaviorally, and how a CHPS intervention might most effectively control them.

## Methods

### Study design

We pursued a qualitative descriptive approach in our study design (Sandelowski, 2000; Doyle et al., 2020). This approach elicits understanding of a phenomenon through the lived experiences of participants, and therefore does not require prior research knowledge, nor conceptual models or frameworks (Sandelowski, 2000, 2010). We chose this approach because of the lack of baseline data in this community regarding the perceived relationship between hypertension and depression, and how to treat these two conditions in practice: the two focal questions of our research. In addition to avoiding assumptions about how to interpret interviews on these questions, qualitative descriptive design also recognizes that these questions are themselves subjective, and might be approached and answered differently by respondents with diverse prior experiences and views (Doyle et al., 2020). Thus, in using this design, we aimed to also capture the unique individual perspectives of participants.

Although we deliberately used no prior conceptual framework to phrase our interview questions, interpret *what* our respondents said about these topics, nor *how or why* they chose to reply as they did, the overall aim of the inquiry was in fact guided by prior research. Specifically, previous data suggest that health providers and community members in rural Ghana would welcome treatment of hypertension and cardiovascular disease through the CHPS model (Haykin et al., 2020; Patil et al., 2023), and that mood and behavior counseling should be a component of this intervention (Wood et al., 2021; Patil et al., 2023). The iterative development and testing of an integrated care model for both hypertension and depression – built to respond to local belief models – is therefore an express aim of this research. To address this aim, we focused our interview guides (see the Supplementary Appendix) around two questions: 1) how these two conditions influence each other in the eyes of local health providers and 2) the most effective and practical CHPS care model to treat them both, separately or together.

### Ethical consideration

We obtained Institutional Review Board (IRB) approval from both the Icahn School of Medicine at Mount Sinai (STUDY-21-01138) and the Navrongo Health Research Centre IRB (STUDY-NHRCIRB250). We obtained oral or written consent from all participants above using previously tested methods (Haykin et al., 2020; Wood et al., 2021).

### Study site, sample and recruitment

We conducted our study in the Kassena-Nankana West and Kassena-Nankana Municipal of the Upper East Region of Ghana. We used convenience sampling to identify CHPS healthcare workers in 13 communities. Specifically, we recruited community health officers (CHOs), CHPS’ frontline primary care providers who provide care at local compounds and perform home visits and community healthcare education; community health volunteers (CHVs), selected community members trained to provide health education to support CHOs; sub-district officers (SDOs) who direct CHPS clinics’ at the sub-district level and district directors (DDs). Additionally, we recruited several community mental health officers (CMHO) who provide basic CHPS mental health care at community and sub-district levels.

### Training of data collectors

We engaged graduate-level research assistants who were staff of the Navrongo Health Research Centre – an implementation science research center of the Ghana Health Service located in the study catchment area with an extensive track record of qualitative research (Haykin et al., 2020; Patil et al., 2023), had at least 1 year experience conducting qualitative interviews and fluent in at least one of the local languages of the study area. Authors R.A.A., E.A., D.J.H. and B.W. introduced the research assistants to the study protocol and instructed them on techniques for asking open-ended questions; clarifying and eliciting further replies through probing; and cultural issues in community entry and consenting as well as protection of confidentiality.

### Data collection

We conducted semi-structured in-depth interviews with community healthcare workers in January and February 2021. Based on the aims above, we designed an interview guide to capture respondents’ understandings of the manifestations of hypertension and depression; how to treat these conditions through CHPS and the perceived linkage between depression and other chronic diseases. Interviewers recorded all interviews, which were translated from Kasem and Nankam to English by trained bilingual staff and then transcribed, using methods previously published by our team (Squires, 2008; Squires, 2009).

### Analysis

We stored, analyzed and transcribed all data verbatim using Dedoose qualitative software (Salmona et al., 2019). We analyzed all data using conventional content analysis, which is a validated and recommended approach for an exploratory or novel research design. Conventional content analysis eschews superimposing pre-existing theory for the creation and application of data codes (Hsieh and Shannon, 2005). Our approach involved generating codes from iterative data review and then applying these codes systematically to the interviews to generate core themes until we reached saturation. First, our research team read each interview word for word and highlighted words that captured key concepts. Next, the researchers reviewed all interview text and made memos of first impressions and initial analysis of codes. Following this process, labels for codes emerged that reflected similar phrases, patterns and relationships across the interviews as a whole. As the process continued, we organized the various codes into categories based on how the codes were related and linked when applied to the text. Then, our team systematically reviewed all fully coded data to identify overarching themes regarding the relationship between hypertension and depression. Specifically, D.A.-A. and M.A.B. performed word-for-word review of transcripts and generated initial codes and categories, which R.A.A., A.P.S. and D.J.H. then independently and reflexively checked and critiqued. Disagreements were resolved initially by initial discussion between D.A.-A. and M.A.B. and then joint review of R.A.A., A.P.S. and D.J.H. Generation of themes and iterative finalization of these results occurred in discussion with these authors and C.R.H. and B.W.

## Results

In total, we interviewed 34 CHPS providers from 13 communities: 11 CHOs, 11 CHVs, 8 SDOs, 2 CMHOs and 2 DDs. Among respondents, 53% were females (unidentified gender = 2), 85% identified as Christian and 15% as other religions. Furthermore, 68% had tertiary education, 16% were senior high school graduates and the other 16% had junior high or primary school education.

Analysis of these 34 CHPS provider interviews revealed three themes regarding explanatory models for how hypertension and depression relate conceptually and how to integrate CHPS care for these diseases. Some respondents posited a unidirectional model in which depression predisposes individuals to developing or worsening hypertension, a relationship mediated by behavioral changes and excessive thinking (Theme 1). Other respondents posited various multidirectional relationships between depression and hypertension (Theme 2). Across nearly all respondents, one’s explanatory model aligned with one’s proposed strategy for treatment (Theme 3).

### Theme 1: Depression as a driver of cardiovascular disease

Many providers felt that depression causes significant behavioral and emotional changes, and these beliefs and actions cause hypertension and other chronic diseases in turn. Of note, these changes took two types: a loss of behavioral control over healthy behavior, causing unhealthy coping behaviors that lead to hypertension; and a new “thinking” that causes depression directly through physiology.

### Loss of control and unhealthy coping strategies

The precise behavioral mediators of depression and hypertension relationship differed among respondents, but nutrition and diet were most common. One CHO explained:

I understand that if you are having mental challenges, you will not be able to control your diet. Diabetes and hypertension are diseases that are all about the ability to control your diet and being able to control your emotions. If you are mentally challenged, you would not be able to do all these things and so, it may worsen other diseases that you may be suffering from including heart disease or diabetes.

Although this CHO described loss of dietary *control* as the means through which depression contributes to a diet that causes chronic disease, another CHO addressed the same question by indicating that:

The fact is that depression comes with a lot of consequences and some may even refuse to eat. Some of the non-communicable diseases like diabetes and hypertension you are talking about needed a regular intake of diet to maintain a healthy body. Now, if someone is depressed and the situation is not well managed, it could potentially influence the person which could lead to complication of the conditions you mentioned.

This CHO posits a different means for dietary change due to depression: loss of appetite that results as a symptom of depression. This behavior pattern, in turn, impacts the nutritional intake necessary for managing chronic diseases such as hypertension. Participants also mentioned how depression influences other behaviors – including alcohol use – which contribute to hypertension. According to one female CHO:

When some of them are depressed, they tend to drink and obviously when you are drinking, it can lead to BP that’s the hypertension.

Earlier in the interview, this CHO noted that individuals with depression “feel that the drink is going to take away whatever they are going through.” Thus, she posits alcohol use as a coping strategy for individuals with depression, but this coping strategy then causes hypertension. Similarly, a CMHO described:

Someone suffering from [depression] will not eat well and may pick up some habits that affect the body negatively. Mentally ill people can drink and smoke anyhow and that can worsen the heart disease and diabetes.

Though the CMHO and CHO identified similar behavioral factors mediating the association between depression and chronic diseases, the CMHO’s description implies a *loss of control* due to depression that led individuals to “pick up some habits” and “drink and smoke anyhow.” In short, mental illnesses like depression cause both active embrace of unhealthy behaviors, and an indifference to one’s behavior, both of which cause hypertension.

The relationship between depression and control – as described by both respondents above – also manifests in how depression impacts care-seeking behaviors such as medication adherence. One CHO stated:

Yes, because, let’s say when you have [depression] you are not yourself so you would not be able to even take care of yourself, even if you are on drugs, you might not remember to even take those drugs because you are not yourself…maybe the person might not also seek healthcare.

The idea of “you are not yourself” again implies decreased self-control or decreased self-awareness as the cause of behavior changes that may worsen chronic diseases.

### Physiological impacts of “excessive thinking”

Participants also narrativized a model in which depression worsens hypertension via thought itself, rather than behavior. Depression was perceived as leading to overthinking and worry, which affects physiology and thereby causes hypertension and other cardiovascular diseases. When asked about whether depression or mental illness can worsen chronic diseases such as hypertension, one CHO replied:

Yes. It can lead to hypertension. Hypertension is about blood pressure and so, when you are always thinking a lot, your heart will be pumping a lot causing your blood pressure to go high. When this happens all the time, it will lead to hypertension and if you are not careful with it, it can lead to stroke. In brief, overthinking can lead to stroke.

This CHO emphasized a physiological connection between thinking and the heart’s function, as “thinking a lot” directly leads to “heart will be pumping a lot” which in turn causes “blood pressure to go high.”

The physiological relationship between excessive thinking and hypertension further emerges in another CHO’s response when discussing depression specifically:

Whatever the cause of the depression is, the person keeps thinking. If you are thinking a lot, you need a lot of blood supply into the various parts of the body. In the process, you can develop a severe form of hypertension. By then, the person would have been withdrawn and doesn’t know what is happening in the surrounding. Because of the extreme nature of the thinking and other factors, it will put a lot of pressure on the blood vessels that are supplying blood to all parts of the body.

While these CHOs differ on whether excessive thinking predisposes or worsens an individual’s hypertension, they share a holistic understanding about the connection between the mind and body. There exists a direct relationship between the impacts of depression on an individual’s psyche and the physiological manifestation of hypertension. In short, “excessive thinking” causes hypertension not through behavior, but through physiology.

### Theme 2: A multilateral relationship between hypertension and depression

Not all participants posited a unidirectional model in which depression causes hypertension through behaviors and thoughts. Two more complex models emerged: some posited a single underlying cause to both conditions, while others posited that both conditions cause each other bidirectionally.

### Underlying cause model

Several participants argued that unhealthy behaviors are the root cause of both hypertension and depression, rather than each causing the other. One CHV said:

When it comes to smoking too, it can let his BP and depression rise, so I think when we advise them and they eat well it reduces the effects of the smoking and will lead to a change in the person.

This respondent expressly described smoking as a common underlying risk factor for both hypertension and depression, rather than a mediator of depression causing hypertension. In contrast, other participants who suggested common behavioral causes of both hypertension and depression did so implicitly. One male SDO described:

And even during durbars, it can be emphasized that when you eat well it helps your health, when you drink plenty it can cause you and when you exercise it improves upon your life. And know when you are fit, you are not exposed to unnecessary stress which may lead you to depression.

Later, when asked about how behavioral counseling could address hypertension, he explained:

You know with hypertension, one of the predisposing factors is stress. And we also have a diet. So, with the diet, counseling people on the way they eat and the way they drink, the extent to which they can eat this or that, then that factor of hypertension will be reduced. Regarding stress, if we are able to educate the people or counsel them on stress and they are living stress-free lives, it will also help to reduce the case of hypertension.

He viewed the behavioral risk factors of diet and alcohol as a common cause for both conditions, though it is unclear whether this participant believed that stress predisposes an individual to one condition over the other – or how stress interacts with risk factors.

### Bidirectional causal model

A few study participants offered a third conceptual model that posited depression and hypertension bidirectionally exacerbating each other. One male MHO described:

One thing that will worsen [chronic conditions] is that, when the person has the mental illness, it means the person will not have that mind to take care of that condition again. … Heart problem that the person is supposed to take precautionary measures, maybe the person will not consider that again, that doctor said I shouldn’t take this and if I do, it may cause me but because of the mental condition, they may not consider that again and may eat anyhow.

Like other participants who cited depression as causing behavioral changes that led to hypertension, he agreed that depression limited an individual’s ability to comply with “precautionary measures” necessary to control cardiovascular issues. However, he later stated:

When we talk about like depression to hypertension, sometimes, physical condition can predispose someone to mental condition especially hypertension. Sometimes when the person hypertension goes to high, it’s like the person is conscience is not there because it’s like the person does not even breathe well so that can affect the brain.

This participant now inverts his prior assertion, describing a physiological mechanism whereby hypertension causes depression because the “person does not even breathe well” thus possibly affecting brain chemistry. This is one of the only physiological causes of depression that respondents mentioned.

Very few participants did not view the two conditions to be linked. When asked if mental illnesses can worsen heart disease, one female CHV replied firmly:

As for me, I don’t think so…Because mental sickness is always based on the brain but for the heart diseases, if you smoke and do other things, that’s where can get it. But for the mental illness, I know it only affects the brain alone.

Unlike the explanatory models in which depression and hypertension are mediated by behavioral factors or bidirectionally impact each other, this respondent believed the causal pathways for the two conditions are distinct. Nevertheless, when questioned further if there is any connection between the brain and the heart, she notes:

Okay, maybe there’s a connection. Sometimes the heart beats and the brain works, maybe if you think about something then your heart beats.

Though uncertain about the connection, she invokes a bidirectional model whereby the two conditions impact each other, and describes a physiological framework in which thinking can affect the function of the heart. Yet her perspective illustrates that not all participants viewed depression and hypertension as linked.

### Theme 3: Disease explanatory models drive proposed health interventions

Regardless of their belief model, nearly all participants volunteered health interventions that would target their perceived root cause of both conditions. For example, participants who favor a unidirectional model where depression leads to hypertension endorsed a treatment approach that focused on breaking that causal pathway, that is, changing behaviors caused by depression to prevent changes in blood pressure. Similarly, those who posit a bidirectional or common cause model favored treatment strategies that align with this viewpoint. When asked if counseling on either depression or hypertension can affect both conditions, one CHV replied:

Absolutely! The solution to depression is counseling. But if you decide not to adhere to the counseling and you go ahead to drink alcohol and do other things, hypertension will continue to rise.

This participant favored the unidirectional conceptual model in which depression leads to hypertension via behavior mediators. Depression counseling must therefore not only treat mood but also prevent alcohol misuse that may worsen hypertension. Another male CHO, who held that depression causes hypertension through excessive thinking, noted:

Managing hypertension in itself is a different matter and so, I am struggling to combine the two. This is because when they are diagnosed by a medical officer and it is established that they have hypertension… they come here for us to monitor their BP daily. But for the depression, maybe, if you want to treat it early so that it doesn’t develop into hypertension and other non-communicable diseases.

Though this respondent initially questions how to integrate care for both diseases, he ultimately embraces his earlier belief that depression can predispose to and worsen hypertension. Therefore, he endorses treating depression early to prevent this development.

Analogously, participants whose conceptual model of the depression–hypertension relationship involved common underlying risk factors endorsed treatment approaches that involved behavioral counseling to control those risk factors. The SDO above who described stress, diet and alcohol use as predisposing individuals to both depression and hypertension also stated:

You know because of ignorance, we involve ourselves in certain lifestyles that cause us a lot. With the drinking you are talking of, it is now the story of the day especially among the youth. But do they have people to tell them the consequences when you drink? So, if there is a counseling unit like that, these people can be brought there and then you give them the counseling.

His focus on “certain lifestyles” – not necessarily on the conditions themselves – aligns with a belief in common behaviors that underlie both depression and hypertension. Similarly, when asked whether depression and hypertension training could be combined, a District Director replied:

Yeah, I think it can be combined. Because for instance, exercise will improve both hypertension and mental health disorders…because most of them it’s about changing behavior and it will help curb both hypertension and depression.

Though the Director’s emphasis is on treatment rather than prevention, both participants emphasized counseling on common behaviors to address both hypertension and mental health disorders.

Finally, participants who posited a bidirectional relationship between depression and hypertension described an approach for treating in conjuction both conditions. For instance, the aforementioned MHO who described depression leading to behaviors that limit chronic disease control, as well as hypertension causing depression through a physiological mechanism, stated:

So, mostly when we have physical condition coexisting with mental condition, mostly, we tackle the physical condition first so that the severity of the physical condition will come down first, then we move in with the mental condition. But if we start with the mental condition, the physical condition will be the predisposed factor, then you are tackling and leaving the case. So, mostly we tackle the physical condition first then when you see that that one is subsiding, then we come with the mental condition. With that, the person will even have time to listen to what you are saying but where someone has hypertension, the person is even restless and you are going to talk of counselling, how would it work.

The MHO supported treating both conditions, based on the view that depression and hypertension could mutually influence each other. From this perspective, both types of treatments will beneficially interrupt this cycle of disease.

## Discussion

Our study explored the perspectives of Ghana’s frontline rural healthcare workers on how depression and hypertension interact casually and behaviorally, and how a proposed primary care intervention could address both conditions. We found that nearly all providers believed these two diseases interact causally. We report three overlapping but distinct explanatory belief models for how depression and hypertension are linked in providers’ view: a unidirectional model in which depressed mood drives hypertension through behavior and physiology, a bidirectional model in which the two diseases cause each other by these means and a common cause model in which common behaviors precipitate both conditions. We also found widespread support for integrated care models for both diseases through Ghana’s CHPS primary care program, which currently offers only limited depression care through specialist providers (Weobong et al., 2023), and hypertension and other NCD care at distant referral centers (Haykin et al., 2020; Wood et al., 2021).

Our findings are significant for two reasons. First, ample evidence suggests that understanding and leveraging the health beliefs and attitudes of key populations is essential to designing effective interventions for chronic disease control in SSA (Sofolahan and Airhihenbuwa, 2012) just as for other health programs targeting underserved populations (Orji et al., 2012; Sheeran et al., 2016). Second, little is known about the beliefs of provider or patient populations in SSA concerning how to treat or prevent cardiovascular diseases (Belue et al., 2009) or depression (Mayston et al., 2020) despite their rising prevalence in this region and across LMICs (Bigna and Noubiap, 2019). Our results contribute to closing this knowledge gap and suggest health provider consensus that both diseases are related to each other through behavioral determinants such as dietary choices and alcohol consumption. Accordingly, health providers propose and endorse treating both conditions with a single intervention. Previous work in rural Ghana has explored health literacy and health beliefs regarding depression (Adu et al., 2021; Adu et al., 2023) but not its relationship with cardiovascular or other diseases, to the best of our knowledge.

Integrating depression screening and care with cardiovascular disease control is also important in LMICs because the two diseases do indeed exacerbate each other (Arokiasamy et al., 2015; Endomba et al., 2020) and evidence from Ghana and elsewhere suggests high rates of comorbidity (Ademola et al., 2019; Amu et al., 2021). However, both diseases are currently undertreated by Ghana’s CHPS primary health system (Laar et al., 2019; Weobong et al., 2023). Few care models in any LMIC have attempted to integrate mental health care with other chronic disease control (Adler et al., 2023) despite emerging LMIC care models in which diseases such as hypertension and diabetes are jointly treated (Vedanthan et al., 2021). Our research therefore aims to inform the creation of a CHPS intervention that could be scalable across the country and beyond, as well as integrated primary care models for mental and other NCDs in similar settings. Insofar as communities and health providers are supportive of integrating care for these diseases in primary care, both directly (e.g., medication for hypertension or depression) and indirectly (e.g., counseling on alcohol reduction to mitigate both conditions), CHPS nurses and health volunteers could be trained to render such care.

Implementation of this model in practice via CHPS will require further study to identify and surmount practical challenges. CHPS nurses are not presently permitted to treat NCDs like depression with medication as a matter of policy, and these agents are not stocked at CHPS compounds. An NCD intervention at the compound level would therefore require rigorous training, careful supervision and transport of essential medications and supplies. However, a telehealth intervention – through which nurses dispense the medication on-site only with the remote approval of an overseeing physician or specialty health officer – could address the first two of these concerns if combined with a teaching and support module. Given that CHPS nurses and volunteers already engage with individuals with NCDs (Haykin et al., 2020), training could emphasize more seamless integration with the primary care and health education already being provided by these healthcare workers. Adding NCD care and counseling duties to CHPS nurses and volunteers’ role could overwhelm their time, but previous data indicate that these providers do not feel that this new role would be onerous (Wood et al., 2021). Furthermore, given that this preventive primary care could mitigate unnecessary hospitalizations and use of higher-cost resources, the Ghana Health Service could hire further CHPS nurses, or provide bonus compensation to these providers or volunteers to ease this burden. The next steps to build on this work should include the pilot testing and quantitative and qualitative evaluation of such a care model for CHPS NCD treatment.

Our study has some notable limitations. While most participants endorsed an integrated disease care model, it is possible this agreement was influenced by how our team represented this concept, as a form of social desirability bias. However, interviewers were trained (as above) in conducting all inquiries in an open-ended unbiased manner, and the interview guide (Supplementary Appendix) aimed to avoid any leading questions or phrases. We focused on only one region of Ghana, and only on some cadres of health workers – our findings, therefore, may not be directly generalized to all regions in Ghana or other countries. Further, though interviews were conducted in Kasem and Nankam languages and English by persons familiar with these languages and associated local cultures, all interviews were translated to English, potentially introducing interpretation gaps related to language and culture. Additionally, not all members of the coding team were fluent in Kasem and Nankam languages, nor were all directly familiar with or residents within the communities under study. However, these weaknesses are counterbalanced by several strengths. We conducted a large number of qualitative interviews, covering a broad range of concepts, and used only highly trained multilingual interviewers in the community to conduct them. Our approach to coding and thematic analysis was systematic and undertaken iteratively by multiple trained researchers, several of whom (R.A.A., E.N. and B.W.) were current or former residents of this community. Further research is indicated to clarify how and whether these findings are reproducible in other contexts and cultures, as well as whether they apply to other mental illnesses or other chronic conditions.

## Conclusion

In a rural setting in Ghana where depression and hypertension are widespread and incompletely treated, we found broad consensus from frontline healthcare workers that these two conditions are causally interrelated, as well as enthusiasm for their integrated treatment in primary care. Our findings suggest that treatment of these and other NCDs through Ghana’s CHPS program – through behavior counseling, medication or otherwise – may be a feasible and culturally acceptable care model worthy of development, testing and scale-up.

## Supporting information

Adu-Amankwah et al. supplementary material Adu-Amankwah et al. supplementary material

## Data Availability

Interview transcripts are not publicly available due to the potential disclosure of information that could compromise the privacy of research participants. However, redacted transcripts are available on request – please contact the corresponding author.

## Supplementary material

The supplementary material for this article can be found at http://doi.org/10.1017/gmh.2024.86.
