# Supplementary material for: Perceptions of healthcare workers on linkage between depression and hypertension in northern Ghana: a qualitative study
Source: Glob Ment Health (Camb). 2024 Oct 10;11:e79. doi: 10.1017/gmh.2024.86 (PMC11504924; doi:10.1017/gmh.2024.86)
Supplement: Adu-Amankwah et al. supplementary material [file S2054425124000864sup001.docx]

**CHO (Community Health Officer) Interview Guide**

We are working to help CHPS treat chronic diseases like high blood pressure and depression. We would like to ask you about care provided both at clinics and on home visits.

This survey is confidential - although we will review your results with our research team and the CHPS staff, this review will be anonymous and will not identify you in any way. You can stop at any time during this interview.

Today we’re going to ask questions mostly about “depression.” We define this as people feeling tension, sadness, severe stress, lack of interest in life. We want to understand how CHPS might be able to treat it.

If someone has these symptoms associated with depression, what might they call it?

How many of your monthly patients do you believe have depression? How do you screen patients for depression, if at all?

What are the causes and risk factors for depression? How might drinking, smoking, poor diet or lack of activity contribute to depression?

What differences between genders have you noticed (for example symptoms, prevalence, stigma)?

What do you think is the best way for CHPS (mental health and primary care) teams to treat depression in your community?

Do you think group therapy or one-on-one counseling would be more feasible? Which one do you think community members would prefer over the other?

Are there adults who would feel uncomfortable discussing depression with CHOs? What about with volunteers?

Are there ways that training could help you understand or care for people with depression? What kind of training would you need (and from whom)?

How do you connect patients with the CHPS mental health teams? What improvements in team coordination are needed to expand mental health treatment?

What kinds of day-to-day challenges exist when treating patients’ mental health?

As you know, we are building a hypertension program, too (can explain further to interviewee, etc.). Do you think that counseling people to change their behaviors (e.g. exercising, drinking, smoking, diet) can be used to address both depression and hypertension?

If we showed you a protocol for this behavior counseling, would you be able to offer feedback? *Add explanation for HAP/CDSMP program*

Would you like to share any experiences that you have had discussing mental health with your patients? With other staff?

How has COVID impacted your ability to carry out your responsibilities?

Have patients shared about their mental health since COVID-19?

How has COVID impacted people’s ability to manage their overall health and chronic conditions?

**Sub-District Officer (SDO) Interview Guide**

We are working to help CHPS treat chronic diseases like high blood pressure and depression. We’d like to understand how nurses (CHOs) and volunteers can treat these conditions.

Today we’re going to ask questions mostly about “depression.” We define this as people feeling tension, sadness, severe stress, or a lack of interest in life.

In your sub-district, how often was depression encountered and reported in [DHIMS](https://www.ghanahealthservice.org/ghs-item-details.php?scid=22&iid=123) in the last quarter?

How do you interact with CHPS mental health nurses?

How should we screen patients for depression?

Do you think that treating depression is a priority in your sub-district?

What do you think is the best way to treat depression in your community?

If CHPS began treating depression with counseling, what type of providers do you envision leading this mental health work in your district?

Do you think group therapy or one-on-one counseling would be more feasible? Do you think that patients would prefer one over the other?

What is volunteer engagement in your subdistrict like? Do you think there is a role for volunteers in delivering this care?

If we showed you a protocol for this behavior counseling, would you be able to offer feedback? *Add explanation for HAP/CDSMP program*

How should we make community members aware of this program (e.g., durbars?)

How are the care teams and mental health teams currently integrated? What changes can be made to best integrate the teams to treat depression?

What does your district’s in-service training for CHO include regarding mental health and risk factors? What is discussed about depression at these sessions?

What kind of training would CHPS nurses need to manage patients’ depression with counseling and medications? Who would provide this training?

What kinds of day-to-day challenges exist when treating patients’ mental health? Barriers might include staffing, space, referrals. How can these barriers be overcome?

For more difficult cases, how can psychiatrists be incorporated into care?

Would you like to share any experiences that you have had discussing mental health with your patients or with other staff?

How has COVID impacted your ability to carry out your responsibilities?

How has COVID impacted people’s ability to manage their overall health and chronic conditions?

Do you anticipate there being a greater need for mental health services after COVID?

**Community Health Volunteer Interview Guide**

Today we’re going to ask questions mostly about “depression.” We define this as people feeling tension, sadness, severe stress, or a lack of interest in life.

***Section 1: CHPS and Current Depression Care***

What do you think depression is? What do you think causes it?

Do you think mental illness can worsen other chronic conditions such as heart disease or diabetes?

Describe your typical day helping out as a CHV for CHPS. How often do you encounter patients you think have depression, and how does it show itself in them?

What challenges do you face in addressing their depression?

What kind of behavior counseling do you offer them for depression?

What kind of work do you do to connect patients with the CHPS mental health nurses?

How is the COVID-19 pandemic influencing people’s mood, and how they work with you?

***Section 2: Potential Solutions to Depression Care through CHPS***

What kinds of depression solutions would work best for your patients?

How might you best work with the mental health and primary care CHOs to treat depression?

What kind of training might you need in order to pursue this work?

***Section 3: Next Steps for Action***

Research shows that some diseases, like hypertension, can be linked with depression. This is because both get worse and better with the same behaviors (like smoking, drinking, or exercise). How could behavior counseling influence these diseases?

Sometimes depression is best managed in a team or group. Would you think it better to help people one at a time, or together?

If we showed you a guide or protocol for how to do this work, in the setting of COVID-19, what feedback could you give us? *Add explanation for HAP program*

Is there anything else you would like to add? (Ask 3 times)
