# Supplementary material for: Perceptions of healthcare workers on linkage between depression and hypertension in northern Ghana: a qualitative study
Source: Glob Ment Health (Camb). 2024 Oct 10;11:e79. doi: 10.1017/gmh.2024.86 (PMC11504924; doi:10.1017/gmh.2024.86)
Supplement: Peer review history

# Editor report: Recommendation: Perceptions of healthcare workers on linkage between depression and hypertension in northern Ghana: a qualitative study — R0/PR2

**DOI:** 10.1017/gmh.2024.86.pr2

**Affiliations:** Psychiatry , Africa Mental Health Research and, Kenya

No accompanying comment.

# Editor report: Decision: Perceptions of healthcare workers on linkage between depression and hypertension in northern Ghana: a qualitative study — R0/PR3

**DOI:** 10.1017/gmh.2024.86.pr3

**Affiliations:** London School of Hygiene & Tropical Medicine, United Kingdom of Great Britain and Northern Ireland

No accompanying comment.

# Editor report: Recommendation: Perceptions of healthcare workers on linkage between depression and hypertension in northern Ghana: a qualitative study — R1/PR5

**DOI:** 10.1017/gmh.2024.86.pr5

**Affiliations:** Psychiatry , Africa Mental Health Research and, Kenya

In note that the authors have extensively revised this paper. I am particularly satisfied about their methodology, their interpretation discussion of the results and their recommendations and limitations. There are very minor typos that can be addressed in a matter of minutes.

# Editor report: Decision: Perceptions of healthcare workers on linkage between depression and hypertension in northern Ghana: a qualitative study — R1/PR6

**DOI:** 10.1017/gmh.2024.86.pr6

**Affiliations:** London School of Hygiene & Tropical Medicine, United Kingdom of Great Britain and Northern Ireland

No accompanying comment.
